# Identification of low-acuity attendances in routine clinical information documented in German Emergency Departments

**DOI:** 10.1186/s12873-023-00838-2

**Published:** 2023-06-06

**Authors:** Anna Slagman, Antje Fischer-Rosinský, David Legg, Kristin Schmieder, Martin Möckel

**Affiliations:** Emergency and Acute Medicine (CVK, CCM), Health Services Research in Emergency and Acute Medicine, Charité Universitätsmedizin Berlin, Augustenburger Platz 1, 13353 Berlin, Germany

**Keywords:** Utilization, Avoidable, Low-urgent, Non-urgent, Emergency department, Primary care treatable, General practitioner treatable, Routine data

## Abstract

**Introduction:**

It has not yet been possible to ascertain the exact proportion, characterization or impact of low-acuity emergency department (ED) attendances on the German Health Care System since valid and robust definitions to be applied in German ED routine data are missing.

**Methods:**

Internationally used methods and parameters to identify low-acuity ED attendances were identified, analyzed and then applied to routine ED data from two EDs of the tertiary care hospitals Charité—Universitätsmedizin Berlin, Campus Mitte (CCM) and Campus Virchow (CVK).

**Results:**

Based on the three routinely available parameters `disposition´, `transport to the ED´ and `triage´ 33.2% (*n* = 30 676) out of 92 477 presentations to the two EDs of Charité—Universitätsmedizin Berlin (CVK, CCM) in 2016 could be classified as low-acuity presentations.

**Conclusion:**

This study provides a reliable and replicable means of retrospective identification and quantification of low-acuity attendances in German ED routine data. This enables both intra-national and international comparisons of figures across future studies and health care monitoring.

**Supplementary Information:**

The online version contains supplementary material available at 10.1186/s12873-023-00838-2.

## Introduction

Although the majority of service users attending Emergency Departments (EDs) in Germany exhibit the genuine life threatening conditions for which these services are designed, there is consistent evidence to suggest that a significant proportion of attendees do not require ED assessment [1–9]. With that said, as there is currently no validated means of retrospectively quantifying and characterising this type of attendance in routine ED data, it has not yet been possible to ascertain the exact proportion or impact of these attendances on the German Health Care System [10].

Accurate quantification notwithstanding, as a recognised challenge in health care systems worldwide [11], the negative impact of these attendances on the provision of urgent care services is widely accepted. In addition to increasing staff workload and impacting care continuity, these attendees can divert resources away from those who need them and increase tension on services already under pressure to deliver high quality and cost-effective care [12–15]. Accordingly, there has been sustained international interest in finding and delivering solutions to this problem.

Germany is no different in this regard. Though the SARS-COV-2 Pandemic saw a significant drop in the number of ED attendances across Germany [16, 17], longer term trends have been of increasing utilisation [18, 19] and it is within this context that service user need for ED resources has become an issue of increasing importance [7]. With this in mind, to best place health professionals and policy makers in Germany to deliver evidenced-based solutions to this problem, it is necessary to develop a reliable and replicable method of retrospective identification and quantification for this type of presentation in routine ED data [20].

To this end, it is first necessary to establish terminology suited to the German Health Care System. For this purpose there are two defining features. First, the system is founded upon a policy universal health care coverage through mandatory health insurance [21] designed to ensure access to affordable and quality care for a majority, regardless of income or status. Second, there is no nationwide system of gatekeeping that regulates access to primary or urgent care services [22]. Access to the health care system in Germany is therefore, in theory at least, independent of the service user’s finances, condition and referral.

Given this structure, and the lack of existing data, this study opts for the term low-acuity to describe ED attendances that received but did not require ED assessment. This term was chosen over alternative existing terminology following the systematic evaluation of their normative connotations, policy implications and functional value relative to the health system [23]. Following the identification, quantification and characterization of these low-acuity presentations considerations can then be made concerning potential policy solutions.

### Research objective

The purpose of this paper is to develop a reliable and replicable method for retrospective identification and quantification of high and low-acuity ED attendances in routine ED data in Germany.

## Methods

### Study design

In order to develop a strategy to identify low-acuity presentations in routine clinical information documented in German EDs, internationally used methods and parameters were identified, analyzed and then applied to routine ED data from two EDs of the tertiary care hospital Charité—Universitätsmedizin Berlin, Campus Mitte (CCM) and Campus Virchow (CVK). The retrospective use of routine data did not require written informed consent and received an ethics vote from the ethics committee of the Charité Universitätsmedizin Berlin (Date of Institutional Review Board (IRB) approval 09.05.2018, IRB-No: EA1_082_18).

### Data collection and management

Routine Clinical ED data were extracted from the hospital information system (HIS) and the ED information system (EDIS) for a one-year period (calendar year 2016). These data were originally documented in the respective systems (HIS, EDIS) by the administrative and medical staff during the patients´ ED consultation. Data from patients younger than 18 years of age at the time of admission to the ED as well as work related accidents were excluded. Data were checked for plausibility and implausible data were corrected or deleted. For this purpose, plausibility cut-offs based on expert consensus were set for all continuous parameters which are listed in Table 1S. Information available in free text fields (mode of transportation to the ED, imaging during ED treatment) were categorized and invalid data, free text corrections and general typos were systematically corrected or deleted.

### Identification of data items to classify low-acuity ED presentations

To develop a suitable and applicable methodological approach to identify low-acuity ED presentations in Germany, a targeted literature review was performed and parameters to identify such presentations were derived and rated regarding suitability within the German health care system and availability in German routine ED data. Furthermore, clinical experts were consulted to identify further data items which might be useful, specifically within in the German health care system that had not been described elsewhere.

Parameters utilized in the international literature to distinguish between high and low-acuity presentations to the ED and which were rated as suitable and applicable to identify low-acuity ED presentations in routine clinical data from EDs in Germany in a retrospective manner are shown in Table 1.

**Table 1:** Data items derived from the scientific literature and expert consens. Respective data values for identification of high and low-acuity presentations are shown

Data item	High-acuity ED presentation	Low-acuity ED presentation
Disposition	admitted or transferred to another hospital	non-admitted
Survival	died	did not die
Mode of arrival to the ED	emergency ambulance/ helicopter/ physician-led medical transport	others or not known
Triage category (MTS, ESI)	1–3	4/ 5 or not known
Glasgow Coma Scale (GCS)	GCS < 15	GCS = 15 or not known
Pain scale (VAS, NRS)	> 5	≤ 5
Respiratory rate per min	< 10 or > 20	10–20
Systolic blood pressure in mmHg	< 90 or > 140	90—140
Body temperature in °C	< 36.3 or > 37.4	36.3 – 37.4
Oxygen saturation in %	< 95	95—100
Heart rate per min	< 60 or > 100	60—100
Imaging performed in the ED	At least one imaging performed in the ED	No imaging performed in the ED
ED-diagnosis	-	list of diagnosis according to Borland [24]

Legend Table 1: Imaging refers to all diagnostic imaging procedures performed in the ED including x-ray, sonography, MRT, CT

*Abbreviation*: *CT* Computer tomography, *ED* Emergency department, *ESI* Emergency Severity Index, *GCS* Glasgow Coma Scale, *MRT* Magnetic resonance tomography, *MTS* Manchester Triage Scale, *NRS* Numeric rating scale, *VAS* Visual analogue scale

Approximately 450 data points were available in the raw data from the documentation system. Those have been excluded on the basis of availability, completeness, quality and expressiveness in the context included: blood values measured in the BGA (blood gas analysis) and laboratory as well as urine; treating department of specialization; service rendered in the emergency room; internally and externally remaining of the patient after emergency room treatment; admitting and discharging department for inpatient treatment; different time stamps, procedures, inpatient secondary diagnoses.

### Statistical analysis and development of a differentiation model

The developed methodological approach to identify low-acuity presentations was then applied in the ED routine data set. A stepwise approach was used to especially allow for an identification method which could handle also missing information within ED routine data, since this is a common problem in German routine ED data. Completeness and validity of the identified data items (Table 2) was investigated in the extracted ED routine data set. Descriptive analyses were performed with SPSS v25.0.

**Table 2:** Completeness of data items in the ED routine data set and data item based proportion of data values to identify high and low-acuity ED presentations

Data item	Completeness whole data set 92 477 (100)	Expression which leads to high-acuity classification	High-acuity ED presentation n (valid %)	Low-acuity ED presentation n (valid %)
Case type	92 475 (100.0)	admitted	23 583 (25.5)	68 892 (75.5)
Referral	37 785 (40.9)	transferred to external hospital	1 729 (4.6)	36 056 (95.4)
Discharge type	23 599 (25.5)	dead	729 (3.1)	22 870 (96.9)
		transferred to external hospital	2 469 (10.5)	21 130 (89.5)
Mode of arrival	78 870 (85.3)	medically accompanied transport	22 436 (28.4)	56 434 (71.6)
Triage	88 589 (95.8)	category: 1–3	49 457 (55.8)	39 132 (44.2)
Glasgow Coma Scale	81 157 (87.8)	< 15	2 186 (2.7)	78 971 (97.3)
Pain-scale:	46 261 (50.0)	> 5	6 784 (14.7)	39 477 (85.3)
Respiratory rate	42 784 (46.3)	< 10 or > 20/min	2 544 (5.9)	40 240 (94.1)
Systolic blood pressure	57 234 (61.9)	< 90 or ≥ 140 mmHg	24 125 (42.2)	33 109 (57.8)
Body temperature	32 038 (34.6)	< 36.3 or > 37.4 °C	11 755 (36.7)	20 283 (63.3)
Oxygen saturation	58 343 (63.1)	< 95%	5 069 (8.7)	53 274 (91.3)
Heart rate	57 814 (62.5)	< 60 or > 100/min	11 303 (19.6)	46 511 (80.4)
Imaging	no specification possible^a^	performed	9 163 (9.9)	no specification possible^a^
ED-diagnosis^b^	80 779 (87.4)	AC-treatable^c^	38 606 (47.8)	42 173 (52.2)

Legend: Absolute and relative proportion of the items considered and the corresponding proportion of presentations with characteristics of high- and low-acuity conditions (absolute and valid percentage)

^a^no information about completeness for these items since information is based on the frequency of documentation of performed information, thus missing information could not be quantified

^b^first documented diagnosis in the ED documentation system

^c^referenced to ambulatory care treatable (AC-treatable) diagnoses Borland et al [24]

## Results

Data of 92 477 presentations to the two EDs of the Charité—Universitätsmedizin Berlin (CVK, CCM) in 2016 were investigated. The mean age was 46 ± 20 years and the proportion of women was 50.3% (*n* = 46 483). The completeness of data items used for identification of low-acuity presentations as well as the corresponding proportion for the high-acuity attendances are shown in Table 2.

**Fig. 1:**
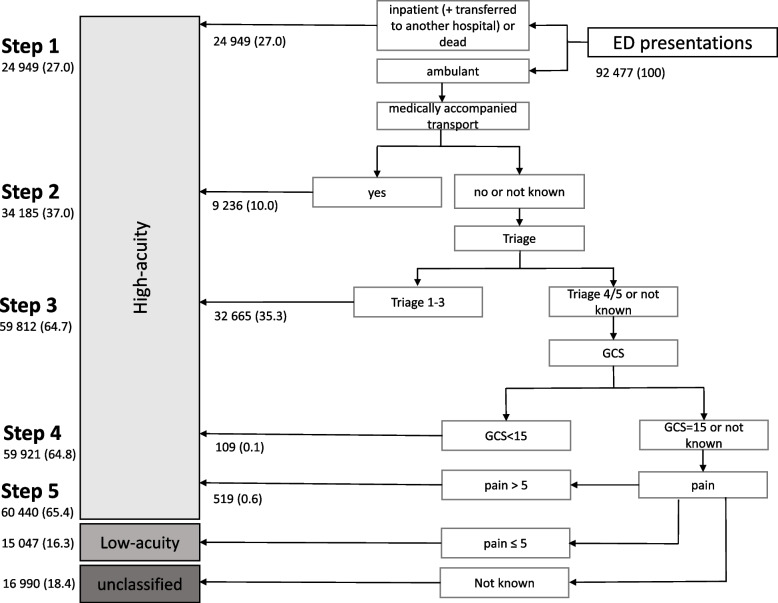
Application of the step-wise approach to categorize high and low-acuity ED presentations: Absolute numbers and percentages (shown in brackets) are illustrated for each individual step and for the final categorization of the total number of presentations. AC – ambulatory care

When the step-wise approach of previously used parameters to identify low-acuity visits to the ED was applied to the actual ED data set (Fig. 1: Step 1–5), 65.4% (*n* = 60 440) of presentations were classified as high-acuity, 16.3% (*n* = 15 047) of all ED presentations were categorized to have been low-acuity and further 18.4% remained unclassified due to missing information (*n* = 16 990).

### Sensitivity analyses regarding further classification of previously unclassified ED presentations

When additional previously identified parameters were considered to further distinguish between high and low-acuity ED presentations within the subgroup of presentations which remained unclassified when the main model was applied (Supplement Fig. 1S), in total 70.7% (*n* = 65 411) of presentations could be assigned to the subgroup of High-acuity ED presentations, 28.7% (26 547) to the subgroup of Low-acuity ED presentations and 0.6% (*n* = 519) remained unclassified.

### Derivation of a final simplified model

**Fig. 2:**
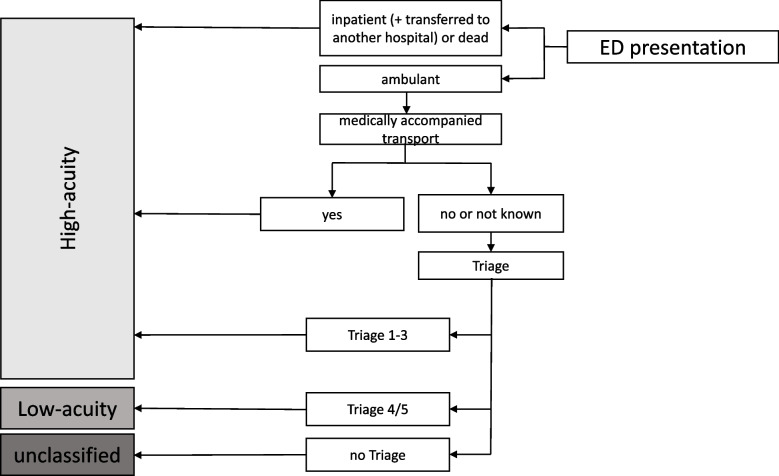
Final step wise model to retrospectively differentiate between high- and low-acuity ED presentations in clinical ED routine data

Considering the results presented above a simplified model was developed and applied in view of the consistent availability and quality of data as well as the proportion of presentations that could be assigned to the target categories based on each data item: This model includes data items which are mainly completely available, show a high data quality and contribute to the assignment of a high proportion of presentations. The final model contains information on ‘survival’, ‘disposition’, ‘mode of arrival’ and ‘triage category’ (Fig. 2). When this final model was applied in the actual ED data set, 64.7% (*n* = 59 812) of presentations were classified as high-acuity while 33.2% (*n* = 30 676) were classified as low-acuity and 2.2% (1 989) remained unclassified.

## Discussion

The aim of this study was to develop a reliable and replicable means of differentiating low-acuity attendances in routine clinical information documented in German EDs. The results indicate that while a majority of ED attendances exhibited the genuine life threatening conditions for which these services are designed, a significant proportion of attendances did not require ED assessment (33.2%). Though these low-acuity attendees may have perceived the need for immediate attention from a medical professional, the systematic criteria utilized in this study indicate that they did not require immediate medical care.

### Interpretation and practical consideration

Though the final figure of low-acuity attendances produced by this study may seem high, within the context of the German Health Care System it is unsurprising. While previous studies have been unable to accurately identify and quantify their impact, the presence and detrimental impact of these Low-acuity presentations in Germany has long been recognised [1–9]. Moreover, given the lack of a nationwide system of gatekeeping regulating access to urgent care services this figure is not unexpected.

To date there remain primarily two ways of accessing health care in Germany. For routine and non-urgent care, service users can access a primary care sector which includes all General Practitioners and Specialists treating service users on an outpatient basis [25]. For urgent care and care outside primary care opening hours, patients can go to a hospital ED or visit ‘Out of Hours’ primary care (OOH) which remains in it’s infancy in Germany [26]. Importantly, the decision to attend any of these services, is in theory at least, unfettered by the service user’s finances, condition and referral.

With that said, lack of gatekeeping and primary care alternatives to the ED alone cannot explain these attendances [27]. While it is beyond the remit of this paper to explain these attendances, attitudinal research has shown that the decision to seek medical help is shaped by a complex web of contributing factors including: service user characteristics; lack of confidence in or access to primary care; perceived need for immediate care; proximity and convenience; and the perceived efficacy of urgent care services [11, 28–31]. This has significant implications for what service users define as a life threatening emergency and maybe at odds with professionally defined health needs, namely, ´the needs for health services as recognised by health professionals from the point of view of the benefit obtainable from advice, preventative measures, management or specific therapy´ [32].

Reasoning aside the results provide clear evidence that these low-acuity attendances represent a huge strain on the German Health Care system. Irrespective of the number of patients in the ED at any given time this type of attendance is detrimental to both the health service provider and user alike. In point of fact, in addition to their impact on individual service users quality of care [12–15], it has been claimed that as each patient covered by state health insurance presenting at the ED results in a loss of 80 euros [21] every low-acuity presentation represents a potentially unnecessary financial burden. Therefore not only healthcare policy changes to assure adequate treatments options for low-acuity presentations to the ED need to be addressed but also the reimbursement of these cases in the ED setting should be reconsidered.

### Comparison of methods to identify low-acuity attendances in the ED

Despite widespread recognition of the detrimental impact of low-acuity ED attendances and long established body of peer review literature [33–35] there remains no universally accepted terminology or definition of this type of ED presentations. Consequently, a number of different methods and criteria have been developed to identify and quantify the impact of this type of attendance leading to significant variation in estimates on both a national and international level [36].

Differences in definition and terminology aside, the exact criteria utilized by individual studies is largely dependent upon the available routine clinical information documented in the ED. As such, a wide range of criteria has been utilized to identify and quantify this type of attendances including; arrival type; comorbidities; diagnoses; hospital admission; presenting condition; referral type; resources used; triage evaluation and vital signs [37]: making comparison of estimates difficult [38]. The exact combination of criteria, and the diversity of approaches which arise from such, should also therefore be understood within the context of differing health care systems.

The data items of the recommended model are in line with current recommendations on ED documentation given by the working group on Emergency Documentation in Germany [39]. These data items are as well included in the current data set of the AKTIN-registry (the German ED routine data registry) [40]. Furthermore they are part of the data items defined as “Notfallkerndatensatz” (NoKeDa [41]) in collaboration of the DIVI-working group and the Robert Koch Institute in Germany [42].

The criteria utilized and promoted by this study are common throughout the literature. In particular, triage evaluation is a key data point in many studies [43–50]. Though these systems alone are an insufficient measure of low-acuity attendances due to the dynamic state of service users’ conditions [51], as a consistent and valuable feature of administrative data [1] when used in tandem with the other data points commonly available in routine ED data such as mode of arrival, admission status or survival [13, 52–56], triage scores provide an important foundation element of many approaches.

Though further information such as hospital resources including imaging, diagnostic tests, procedures, or medications ordered are also used to identify low-acuity attendances [12, 57, 58] this information is not widely available in routine ED data in Germany and furthermore the availability of these resources in the primary care setting varies across locations and settings and could as well be improved in the future and thus would not necessarily require treatment in an ED.

### Strength and weaknesses of the study

As the first study to provide a reliable and replicable method for accurately identifying and quantify the proportion of low-acuity attendances across urgent care services in Germany, this study provides a vital tool for the advancement of targeted and evidence-based policy solutions in the German Health-Care system. Indeed, to date, no study has been able to accurately quantify and characterise attendees who do not require ED assessment. In providing a method for doing so, this study enables the furthering of patients-centered health care for high and low-acuity patients alike.

Furthermore, while the impact of low-acuity attendees is widely accepted, the direct impact of low-acuity attendances remains under-researched. By establishing an accurate method of identification and quantification, this study paves the way for future research into the direct impact of low-acuity attendees on the provision of urgent care services. For example, while much has been made of the relationship between low-acuity attendees and ED overcrowding, evidence of their relationship to one another is often over-stated [59]. By accurately identifying which patients require and do not require ED assessment, the method detailed in this study provides a means of measuring their direct impact.

Potential gains aside, it should be noted that as ED documentation in Germany remains unstandardized and electronic documentation is not yet implemented comprehensively [60], data items that could not be considered in this study due to data availability and quality could have further improved this method of classification. As it stands, the current method makes use of the most widely available clinical data points enabling the highest number of potential applications across the German Health System and internationally.

As for the items included in the final algorithm, each individual data item has potential limitations. For instance, it could be argued that “Medically accompanied transport” is an insufficient marker of patient’s acuity given that not every patient transported by ambulance will necessarily require ED treatment. The combination on the other hand, medically accompanied transport offers a widely and readily available item, anchored in international algorithms and, at least to a limited extent, is a indicator of acute urgency for treatment.

Also regarding the triage systems ESI and MTS it has to be mentioned that they are not 100% comparable. For this pragmatic approach they were and should be handled equally. The use of more objective parameters like vital signs and NRS-score, have not led to any improvement in assignment (sensitivity analysis – vital signs, diagnoses, imaging 1S). Furthermore, these data have a much poorer quality and availability, which makes their application in a pragmatic easy-to-transfer approach for routine ED data not reasonable but they could be evaluated for prospective identification of low-acuity patients without any doubt.

The above notwithstanding, it is important to keep in mind that the presented final algorithm is not intended to prospectively identify low-acuity patients but to give a retrospective estimate of the proportion of low-acuity cases in the ED. As such it cannot be used to predict the number of low acuity presentations. Instead, it should be utilized for the development of targeted solutions and monitoring time trends relative to the impact of demographic changes, interventions and public policy solutions.

## Conclusion

This study provides first accurate estimates of low-acuity attendances in EDs in Germany (33,2%) and a reliable and replicable means of retrospective identification and quantification of these attendances in German ED routine data. This enables both intra-national and international comparisons of figures across future studies and health care monitoring. Since estimates of low-acuity attendances are likely to vary between different EDs further analyses in a more heterogeneous sample of German EDs are required.

## Supplementary Information

**Additional file 1: Table 1S.** Plausibility cut-offs defined by expert consensus for continuous parameters routinely documented in the ED.

**Additional file 2: Figure 1S.** Sensitivity analysis regarding further classification of previously unclassified ED-presentations. AC – ambulatory care, CT – computer tomography, MRT – magnet resonance tomography.

## Data Availability

The dataset analysed during the current study is not publicly available due to the high sensitivity of clinical data of the patients in the emergency department but are available from the corresponding author on reasonable request, if necessary in aggregated entity to ensure that no identification is possible**.**

## Abbreviations

AC-treatable: Ambulatory care treatable
AKTIN: The German ED routine data registry
BGA: Blood gas analysis
CCM: Charité Universitätsmedizin Berlin, Campus Mitte
CVK: Charité Universitätsmedizin Berlin, Campus Virchow
DIVI: Deutsche Interdisziplinäre Vereinigung für Intensiv- und Notfallmedizin (German Interdisciplinary Association for Intensive Care and Emergency Medicine)
ED: Emergency departmend
EDIS: Emergency department information system
ESI: Emergency severity index
GCS: Glascow coma scale
HIS: Hospital information system
IRB: Institutional Review Board
MTS: Manchaster triage system
NoKeDa: Notfallkerndatensatz (Core Dataset "Emergency Department")
NRS: Numeric rating scale
OOH: Out of hours
VAS: Visual analogue scale
MRT: Magnetic resonance tomography
CT: Computer tomography
