# Supplementary material for: Identification of low-acuity attendances in routine clinical information documented in German Emergency Departments
Source: BMC Emerg Med. 2023 Jun 6;23:64. doi: 10.1186/s12873-023-00838-2 (PMC10243890; doi:10.1186/s12873-023-00838-2)
Supplement: Supplementary file 1 — Additional file 1: Table 1S. Plausibility cut-offs defined by expert consensus for continuous parameters routinely documented in the ED. [file 12873_2023_838_MOESM1_ESM.docx]

Table 1S: Plausibility cut-offs defined by expert consensus for continuous parameters routinely documented in the ED.

| **Parameter** | **Range of plausible values** |
| --- | --- |
| Respiratory rate (breaths/min) | 0 - 100 |
| Heart rate (beats/min) | 0 - 300 |
| Systolic blood pressure (mmHg) | 0 - 300 |
| Body temperature (°C) | 15 - 45 |
| Oxygen saturation (%) | 0 - 100 |
| Pain score (VAS or NRS) | 0 - 10 |
| Glasgow coma scale | 3 - 15 |
| Manchester triage category (MTS) | 1 - 5 |

Legend Table 1: Abbreviation: MTS – Manchester Triage Scale, NRS – numeric rating scale, VAS – visual analogue scale
