# Supplementary material for: Identification of low-acuity attendances in routine clinical information documented in German Emergency Departments
Source: BMC Emerg Med. 2023 Jun 6;23:64. doi: 10.1186/s12873-023-00838-2 (PMC10243890; doi:10.1186/s12873-023-00838-2)
Supplement: Supplementary file 2 — Additional file 2: Figure 1S. Sensitivity analysis regarding further classification of previously unclassified ED-presentations. AC – ambulatory care, CT – computer tomography, MRT – magnet resonance tomography. [file 12873_2023_838_MOESM2_ESM.docx]

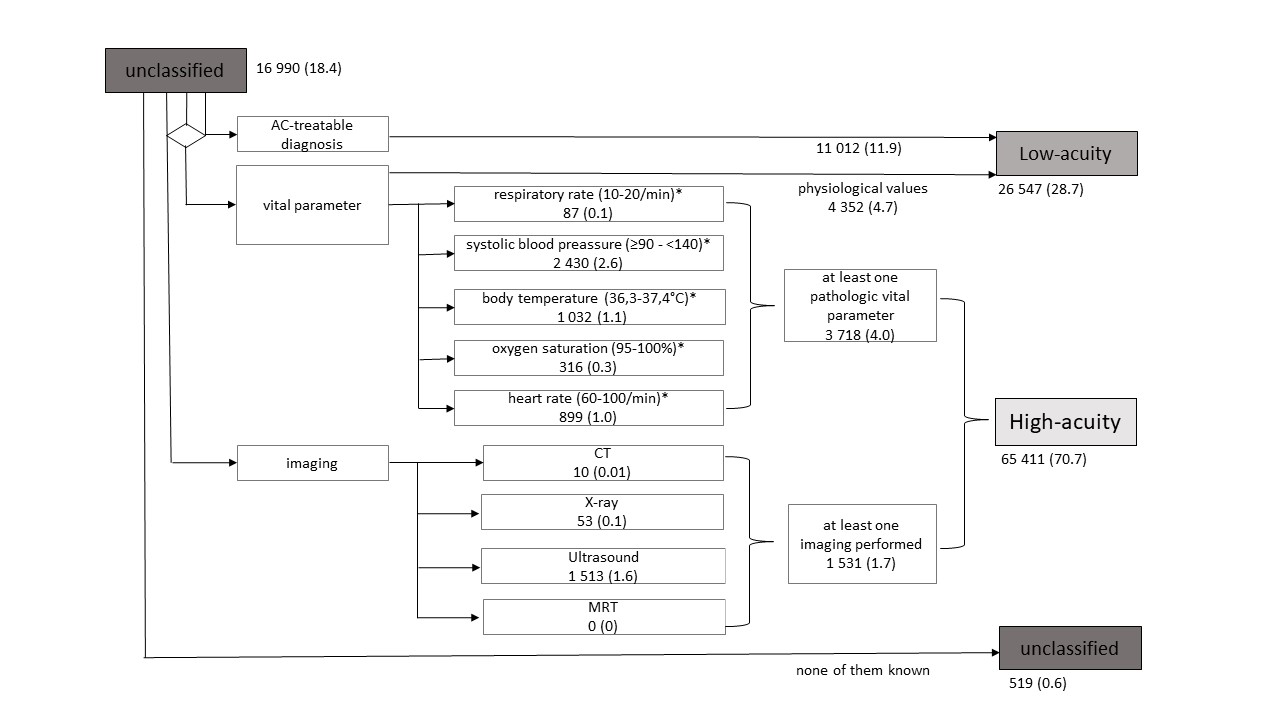


Figure 1S: Sensitivity analysis regarding further classification of previously unclassified ED-presentations. AC – ambulatory care, CT – computer tomography, MRT – magnet resonance tomography.
